# Performance of the intracerebroventricularly injected streptozotocin Alzheimer’s disease model in a translationally relevant, aged and experienced rat population

**DOI:** 10.1038/s41598-022-24292-5

**Published:** 2022-11-24

**Authors:** Attila Gáspár, Barbara Hutka, Aliz Judit Ernyey, Brigitta Tekla Tajti, Bence Tamás Varga, Zoltán Sándor Zádori, István Gyertyán

**Affiliations:** Department of Pharmacology and Pharmacotherapy, Semmelweis University, Nagyvárad tér 4, Budapest, 1089 Hungary

**Keywords:** Diseases of the nervous system, Alzheimer's disease

## Abstract

The intracerebroventricularly (icv) injected streptozotocin (STZ) induced brain state is a widely used model of sporadic Alzheimer-disease (AD). However, data have been generated in young, naive albino rats. We postulate that the translationally most relevant animal population of an AD model should be that of aged rats with substantial learning history. The objective of the study was thus to probe the model in old rats with knowledge in various cognitive domains. Long-Evans rats of 23 and 10 months age with acquired knowledge in five-choice serial reaction time task (5-CSRTT), a cooperation task, Morris water-maze (MWM) and “pot-jumping” exercise were treated with 3 × 1.5 mg/kg icv. STZ and their performance were followed for 3 months in the above and additional behavioral assays. Both STZ-treated age groups showed significant impairment in the MWM (spatial learning) and novel object recognition test (recognition memory) but not in passive avoidance and fear conditioning paradigms (fear memory). In young STZ treated rats, significant differences were also found in the 5CSRTT (attention) and pot jumping test (procedural learning) while in old rats a significant increase in hippocampal phospho-tau/tau protein ratio was observed. No significant difference was found in the cooperation (social cognition) and pairwise discrimination (visual memory) assays and hippocampal β-amyloid levels. STZ treated old animals showed impulsivity-like behavior in several tests. Our results partly coincide with partly deviate from those published on young, albino, unexperienced rats. Beside the age, strain and experience level of the animals differences can also be attributed to the increased dose of STZ, and the applied food restriction regime. The observed cognitive and non-cognitive activity pattern of icv. STZ in aged experienced rats call for more extensive studies with the STZ model to further strengthen and specify its translational validity.

## Introduction

Development of animal models with better translational relevance is essential for better understanding of Alzheimer’s disease (AD) and for more efficient drug development as well. Regrettably, no new cognitive enhancers have been found in the last 20 years mostly due to lack of efficacy^1,2^. Disease modifying drugs most advanced in the pipeline—but finally failed—targeted the β-amyloid cascade^3^ and relied on transgenic mouse models of the familial form of the disease^4^. The Intracerebroventricularly (icv) injected streptozotocin (STZ) represents an alternative approach as it is a widely used model of sporadic AD. The construct validity of the icv. STZ model is based on the induced insulin resistant brain state^5^ which gives rise to many symptoms of AD, such as cognitive deficiency and increased phospho-tau at 1 month post-injection, elevated β -amyloid level at 3 months, appearance of plaques at 6 months^6,7^.

Our group established a rodent test system in which the animals acquire several types of cognitive tasks and then maintain their performance in regular training sessions^8–10^. Hereby we create a population with “widespread knowledge” which better models the human population than naïve or freshly taught animals. This “widespread knowledge” is then impaired with various kinds of interventions to create a ‘patient population’, amenable to test cognitive enhancers on. We use Long-Evans rats as experimental subjects because of their well-known good learning ability, which is an essential requirement in the system^11–15^. Integration of the STZ induced insulin resistant brain state model to our specific test system could result in a model which well imitates the human cognitive decline.

As the icv. STZ model has been almost exclusively used in young albino rat strains, in our previous experiments, we already examined the effect of STZ in young naïve Long-Evans rats and found that an increased dose was required to elicit subtle AD-like symptoms^16^. These results suggest that there may be specific differences between strains.

In this study, we examined the effect of icv. STZ in Long-Evans rats with widespread knowledge in two different age groups (old and young), since theoretically, old experienced animals are translationally the most relevant population for the experimental investigation of AD. For logistical reasons the two age groups were studied in two separate experiments.

## Methods

### Animals

Twenty-nine 23 months old and twenty-four 10 months old male Long-Evans rats (‘old’ and ‘young’ animals, respectively; obtained from Janvier Labs, Le Genest-Saint-Isle, France) were used in this study. Animals were kept three in a cage with paper tubes and wooden bricks as environmental enrichment tools under reverse light dark cycle (dark phase from 4 am to 4 pm). Animals had a restricted food access: 45 g of food was supplied for three rats before the end of the dark phase. We kept the animals under this regime because food restriction has repeatedly been shown to be healthier than *ad lib* feeding, slow the aging process and the age-associated increase in mortality rate^17–20^ as well as prolong cognitive functioning^21–23^. Furthermore, this regime made the animals motivated to work in the food-rewarded tasks on the following day. Food restriction was suspended for the period of icv. STZ injections and one week recovery thereafter when rats had free access to food*.* Drinking water was available ad libitum over the whole course of the experiment. The animals were intensively handled before and during the experiments and were regularly trained in several learning paradigms for 21 months (old animals) or 8 months (young animals), these are specifically described below and in the Supplementary material. At the end of the post treatment behavioral measurements, they were anaesthetized by isoflurane and decapitated to remove their hippocampus for the western blot measurements. The experiments were authorized by the regional animal health authority in Hungary (resolution number PE/EA/85–5/2019) and conformed to the Hungarian welfare law and the EU 63/2010 Directive and ARRIVE guidelines.

### Experimental design

**Figure 1:**
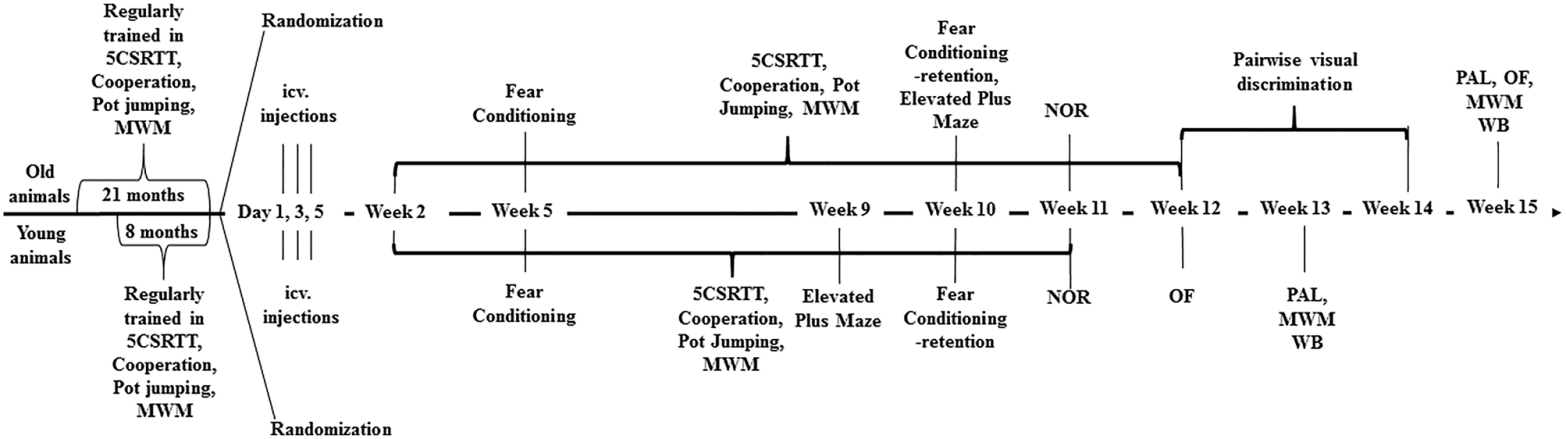
Timeline of the experiments (*icv* intracerebroventricular, *STZ* streptozotocin, *NOR* novel object recognition, *5CSRTT* five choice serial reaction time task, *OF* open field, *PAL* passive avoidance learning; *MWM* Morris water-maze, *PD* pairwise visual discrimination, *WB* western blot).

The flow of the experiments is shown in Fig. 1. Sample size determination for young rats was carried out by power analysis centered on the novel object recognition test since it has got the largest standard deviation among the behavioral assays. We obtained values from the G*Power 3.1.9.7 software^24^, (n = 12) as the group size for young animals. From the available 29 old animals we assigned 15 to the control and 14 to the STZ group taking into account possible losses because of their age. Based on the baseline results in the cognitive assays the animals were randomly assigned to the treatment groups (STZ or vehicle) (Fig. 1). In the experiment with the old animals, two STZ-injected and three control rats did not recover from anesthesia. We lost four additional animals from the STZ group in the course of the experiment. Two died at weeks 2 and 11, while two others were euthanized due to poor health at weeks 9 and 11.

### Intracerebroventricular streptozotocin treatment

Icv. injection of STZ was carried out according to Gáspár et al.^16^. 4.5 mg/kg STZ (Sigma-Aldrich, St. Louis, MO, United States) split into three equal doses (1.5 mg/kg) was administered on days 1, 3, and 5. A volume of 2μL/ventricle was injected to the left and the right ventricle for a rat of 500 g. The dose was adjusted to the body mass of the animal by changing the injection volume. STZ was dissolved in 0.05 M citrate buffer pH 4.5 (Santa Cruz Biotechnology, Santa Cruz, CA, United States). The control groups received vehicle treatment in both experiments. Rats anaesthetized via a mixture of ketamine (80 mg/kg) (Produlab Pharma B.V. Raamsdonksveer, Netherlands) and xylazine (10 mg/kg ip.) (Produlab Pharma B.V. Raamsdonksveer, Netherlands) during the first drug administration and isoflurane (4% in pure oxygen) (CP-Pharma GmbH, Burgdorf, Germany) during the 2nd and 3rd surgeries. The icv. coordinates were: 0.72 mm posterior to bregma, 1.5 mm lateral to sagittal suture, 3.6 mm ventral of the surface of the brain^25^.

### Behavioral assays

#### Morris water-maze (MWM)

The apparatus^26^ was a black circular pool (diameter 190 cm, depth 60 cm) filled with water (38 cm, 23 ± 1 °C) and containing a non-visible round escape platform (10 cm diameter) placed 0.5 cm below the water surface. The platform was located in one of the four quadrants (south-east (SE), south-west (SW), north-east (NE), north-west (NW)), 40 cm from the edge of the pool. On the wall of the experimental room extra-maze cues were placed to facilitate the orientation during swimming. The learning session consisted of 3 daily trials. At the start of a trial the rat was placed into the pool at one of the four possible start points and had 3 min to find the hidden escape platform. When the animal didn’t find it, it was gently guided to the platform and allowed to climb onto it. Rats could spend 30 s on the platform then were taken out, dried and replaced in their home-cage. The interval between the trials was 30 min. Escape latency was measured and swimming path was recorded by Smart v3.0 video tracking system software (Panlab, Barcelona, Spain). Rats learned the task with the platform fixed at the SE quadrant, then they received monthly maintenance training sessions in which the location of the platform was rotated around the four quadrants from session to session.

#### 5-choice serial reaction time task (5CSRTT)

The 5CSRTT device^27^ consisted of a 31 × 35 × 34 cm test box (cat. no. 259920) (TSE Systems, Bad Homburg vor der Höhe, Germany). The boxes were equipped with 5 nose-poke modules on the back wall and with a magazine at the front wall. During the task, after 5 s inter-trial interval, in one randomly selected nose-poke module a 1 s long stimulus was presented and the animal had to nose-poke into the signalled hole. The animal made a correct response if nose-poked into this hole during the stimulus presentation or within 5 s afterwards (limited hold). Correct responses were rewarded with a pellet delivered into the magazine. Nose-poke into the magazine initiated the next trial. The animal made an incorrect response if nose-poked into one of the holes where the stimulus was not presented. An omission response was recorded when the rat did not make any nose-poke up to the end of the limited hold. Incorrect responses and omissions were followed by 5 s time-out punishment, when the house light was turned off. After the time-out, the house light was set back and the rat could start the next trial by nose-poking into the magazine. The animal made a premature response, if nose-poked into any of the holes during the inter-trial interval. These responses were also punished with time-out. Length of a daily test session was 20 min. The outcome parameters were the percentages of correct, omission and premature responses and accuracy defined as $\left(\frac{total\, correct\, responses}{total\, correct\, responses\, +\, total\, incorrect\, responses}\,\times\,100\right)$.

#### Pot jumping

The experiment was conducted according to Ernyey et al.^28^. In the MWM tank 12 flower pots (16 cm high and 10 cm wide at the bottom) were placed upside down forming a circle. Distance between the centers of the adjacent pots gradually increased from 18 to 46 cm in anticlockwise direction. The tank was filled with 6 cm deep water to restrain rats climbing off the pots. During a session, animals were placed onto the start pot, which was within the shortest distance from the next pot. For 3 min they could freely move on the pots and their behavior was observed and recorded with a video camera system. Outcome parameters were the longest interpot distance jumped over and the number of passes.

#### Cooperation task

Social memory was measured in a cooperation task modified after Kozma et al.^29^. in a 30 × 24 × 21 cm Skinner box (MedAssociates, VT, USA). The opposite walls of the chamber were equipped with one nose-poke module, one lever press module and one magazine for each. During the task, the animals worked in pairs but were separated from each other by a separating fence. One of the animals had to nose poke in to the nose poke module for 3 s, when it activated the lever press module at the opposite side. The other animal had to push the lever, as a result of which they received a reward pellet and started a new trial. The task was unsuccessful if one of the steps was missing. An omission response was recorded when the rats did not make any nose-poke or lever press. Out of sequence and incorrectly timed responses were punished with 5 s timeout. Length of a daily test session was 20 min.

#### Fear conditioning

The test device was a sound-proof shocking chamber (26 × 26 × 30 cm) (Ugo Basile, Gemonio, Italy) in which the fear-behavior of the animals was recorded with an infrared video camera controlled by the software EthoVision v13.0 (Noldus, Wageningen, Netherlands). The experiment, based on Varga et al.^30^, consisted of one acquisition and two retention trials (24 h and 1 month later). Duration of each session was 5 min. During the acquisition trial, the rats received 5 mild foot-shocks as unconditional stimulus (0.6 mA, 1 s), the delay between shocks was 60 s. The shocks were preceded by a combination of continuous sound (65 dB, 3 kHz) and flickering light (1 Hz) conditional stimuli for 10 s, in the last second overlapping the unconditional stimulus. During retention trials, the animals received the same conditional stimuli, in absence of the foot shock. The main outcome variable was the animals’ freezing time.

#### Novel object recognition (NOR)

The test apparatus^31^ was a 48 × 48 × 42 cm box with bedding material on the bottom where the behavior of the animals were recorded by a video camera system. The assay consisted of an acquisition trial and a retention trial. In the acquisition trial, the rats had 3 min to explore two identical objects in the box. After a delay of 80 min, in the retention trial one of the objects was changed to a novel one and the animals had 3 min again to explore them. The recognizable objects were a plastic bottle filled with gravel and a glass bottle filled with blue dye solution. Exploration time of each object was the registered parameter. Recognition memory was characterized by the discrimination index, DI = $\frac{new\, object-old\, object}{new\, object+old\, object}$. Rats that explored the objects for less than 10 s or explored only one of the two objects in any of the trials were excluded from the evaluation (one animal from the control group and one rat from the STZ group among the young animals).

#### Pairwise visual discrimination

The task^32^ was carried out in a touchscreen apparatus (Campden Instruments Ltd., Lafayette, IN, USA, cat. no. 80604). The boxes were equipped with a touch screen at the front and with a magazine at the back wall. The touchscreen wall can be divided into two sections using a cover panel. Subjects (old animals) were trained to discriminate between two images (one was correct, the other was incorrect) presented randomly in the left or right window of the touchscreen. Nosepoking the correct image was rewarded with a pellet. Choosing the incorrect image led to 5 s time out, when the houselight turned on. Entering and exiting the food magazine initiated the next trial i.e. appearance of the two images. Length of a daily test session was 30 min. Number of completed trials, correct and incorrect responses were registered by ABET II touch v2.15 software.

#### Passive avoidance learning (PAL)

The type of the experiment was a step through passive avoidance test^33^. The apparatus consisted of a light and a dark chamber separated by a guillotine door. The test consisted of two parts, the acquisition trial and 24 h later the retention trial. During the trials the rats were placed into the light chamber and 30 s later the door opened and the animal could cross into the dark chamber. In the acquisition trial the animals had 180 s (cut off time) to enter the dark compartment of the device, whereas at the retention trial the cut off time was 300 s. When the rat passed through to the dark side, the door closed and after a 3 s delay a mild foot shock (0.6 mA, 3 s) was delivered. The animal was left in the dark compartment for an additional 5 s after the shock. The measured parameters were entry latencies into the dark compartment in the acquisition and the retention trials. Animals which did not cross to the dark chamber at the acquisition trial were excluded from the experiment (two rats from the STZ group in the young group).

#### Elevated plus maze (EPM)

The apparatus^34^ consisted of four arms (50 × 15 cm), two opened and two closed arms, the latter with 40 cm high walls. The arms were connected in a central square (15 × 15 cm). The entire maze was elevated 52 cm from the floor. The animals were placed in the central square, facing one of the open arms and had 300 s to explore the maze. The behavior of the rats were recorded by a video camera system. The measured parameters were the times spent in the open arms and the entry numbers to the arms. One rat from the young STZ group which did not moved from the central square was excluded from the experiment.

#### Open field (OF)

The test apparatus^35^ was a 48 × 48 × 40 cm box with 30 × 30 infrared beam net where the horizontal and vertical behavior of the animals were recorded by automated Conducta moti-meter system (Experimetria, Budapest, Hungary). The animals placed in the center of the apparatus and their behavior was recorded for 30 min. Analyzed parameters were the ambulation time, local movement time and immobility time.

### Western blot (WB)

After the behavioral tests, the animals were decapitated, their brains were removed and both hippocampi were dissected then frozen and stored at − 80 °C. Membranes were incubated with primary antibodies (obtained from Santa Cruz Biotechnology, Santa Cruz, CA, United States) against Phospho-Tau (p-tau) (PHF-13, sc32275)^36^, Tau (sc32274)^37^ and β-Amyloid (sc28365)^38^ overnight at 4 °C, followed by 2 h incubation at room temperature with anti-mouse HRP-linked secondary antibody. Phospho-Tau protein expression was normalized to the corresponding total protein. β-actin was used to control for sample loading and protein transfer and to normalize the content of the β-amyloid.

### Data and statistical analysis

Group means ± standard error were calculated and significance was determined by unpaired t-test (NOR, PAL, EPM, OF, WB), single sample t-test (NOR), repeated measures ANOVA (MWM, pot jump, 5CSRTT, cooperation, fear condition, pairwise visual discrimination) or one-way ANOVA (WB) using the Statistica 13.5.0.17 software package (TIBCO Software Inc.). In tasks involving repeated measurements, data of animals lost (died or euthanized) during the course of the experiment were handled according to the last observation carried forward method. The number of old animals actually taking part in a measurement is shown in the corresponding figure legend.

## Results

### Morris water-maze (MWM)

**Figure 2:**
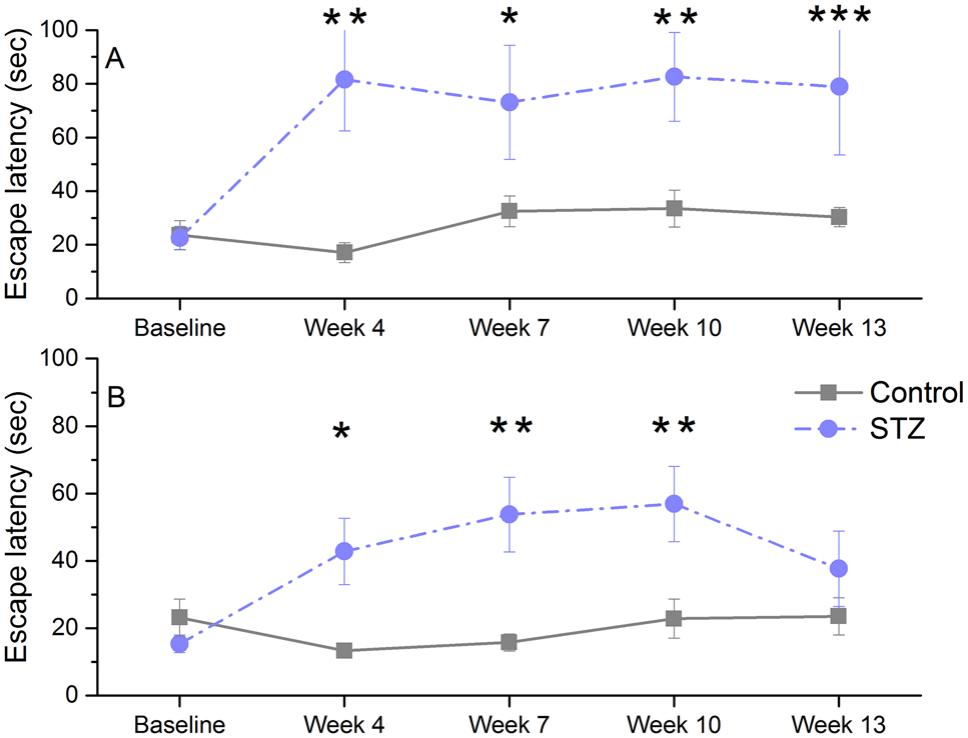
Learning performance of icv. STZ-injected (‘STZ’) and vehicle-treated (‘control’) rats in the Morris water-maze at various time points post-injection. Means ± SEM of daily latency values are shown. (**A**) Results of old rats. *, **, ***: *p* < 0.05, *p* < 0.01 *p* < 0.001: significant difference between groups on days 1 2, 3 and 4 (post-hoc Duncan test following repeated measures ANOVA with significant Day × treatment interaction: F(4, 84) = 6.13, *p* = 0.000). (**B**) Results of young rats. *, **: *p* < 0.05, p < 0.01: significant difference between groups on days 1 2 and 3 (post-hoc Duncan test following repeated measures ANOVA with significant Day × treatment interaction: F(4, 88) = 3.88, *p* = 0.006). Group size of old STZ-treated rats: n = 11 at Week 4 and 6–8, n = 10 at Week 9–11, n = 8 at Week 14–15.

STZ-treated rats needed significantly longer time to find the hidden platform in both experiments. The difference was maintained throughout the whole measurement period except at day 4 in young rats, when the treated animals performed similarly to controls though still significantly worse than at their own baseline (Fig. 2A,B).

### 5-choice serial reaction time task (5CSRTT)

**Figure 3:**
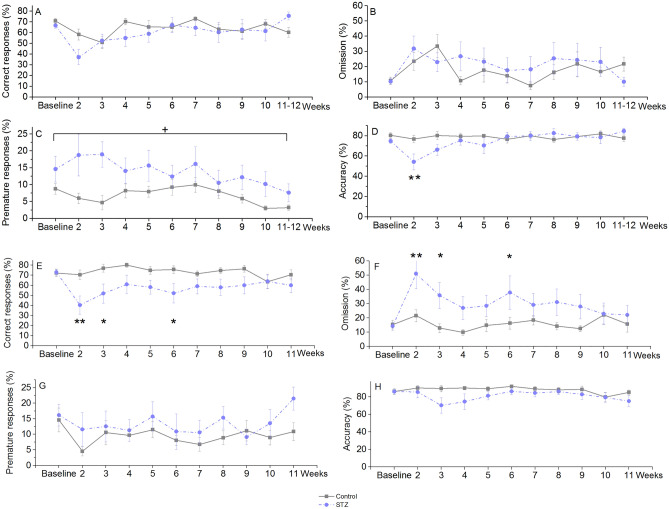
Learning performance of icv. STZ-injected (‘STZ’) and vehicle-treated (‘control’) rats in the 5CSRTT at various time points post-injection. Means ± SEM values are shown. (**A**, **B**, **C**, **D**) Results of old animals. + : *p* = 0.023 significant treatment effect in percentage of premature responses (F(1, 21) = 5.98). **: *p* < 0.01 significant difference vs control on the same day (post-hoc Duncan test following repeated measures ANOVA with significant Day × treatment interaction: F(10, 200) = 2.53, *p* = 0.007) for percentage of accuracy. (**E**, **F**, **G**, **H**) Results of young animals. *, **: *p* < 0.05, *p* < 0.01 significant difference between groups on the same day (post-hoc Duncan test following repeated measures ANOVA with significant Day × treatment interaction: F(10, 220) = 2.20, *p* = 0.019 for percentage of correct responses and F(10,220) = 2.06, *p* = 0.029 for omissions). Group size of old STZ-treated rats: n = 12 at Week 2, n = 11 at Week 3–9, n = 10 at Week 10, n = 8 at Week 11–12.

Old STZ-treated rats produced significantly more premature responses than that of the controls in the post-injection period from Week 2 to Week 12 (Fig. 3C). There was no significant difference between the groups in the percentage of correct responses and omissions (Fig. 3A,B). Response accuracy was significantly lower in the ‘STZ’ group on the first post treatment occasion, however, this difference was not detectable on additional measurement days (Fig. 3D). In young rats, STZ-treated animals showed significantly reduced correct responses and increased omissions up to Week 6 (Fig. 3E,F) with preserved accuracy (Fig. 3H) and unchanged premature nosepokes, except in the very last session (Week 11), when the latter was elevated compared to controls (Fig. 3G).

### Pot jumping

**Figure 4:**
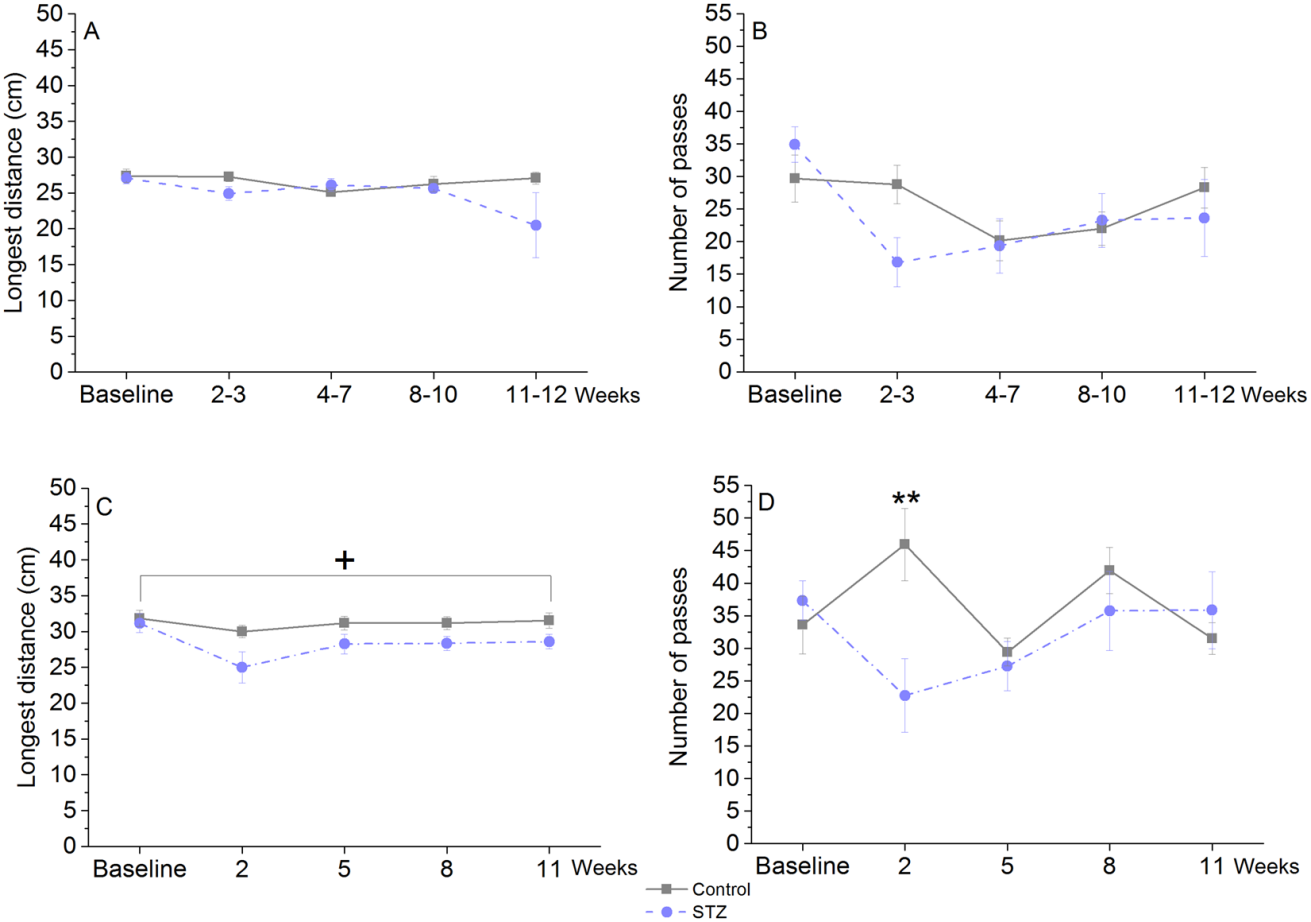
Performance of icv. STZ-injected (‘STZ’) and vehicle-treated (‘control’) rats in the pot jumping task at various time points post-injection. Means ± SEM of number of passes and longest distance jumped over are shown. (**A**, **B**) Results of old animals. (**C**, **D**) Results of young animals. **: *p* < 0.01 significant difference between groups on the same day (post-hoc Duncan test following repeated measures ANOVA with significant Day × treatment interaction: F(4,88) = 5.20, *p* = 0.000. + : *p* = 0.047 significant treatment effect in longest distance (F(1, 22) = 4.42). Group size of old STZ-treated rats: n = 11 at Week 2–3, 4–7 and 8–10, n = 8 at Week 11–12.

In old rats, we could not detect significant difference between the groups in this procedural learning task either in the longest interpot distance jumped over or in the number of passes (Fig. 4A,B). In contrast, young STZ-injected rats jumped over significantly shorter distance than control rats, and made significantly less passes at the first post-treatment occasion (Week 2) (Fig. 4C,D).

### Cooperation

In old rats, because of the high mortality rate the pairs were broken and it was not possible to evaluate the data. In young rats, there was no significant difference between the learning performances of the two groups (Fig. S1).

### Fear conditioning (FC)

There was no significant difference between the behavior of the animals in acquisition trials in any of the experiments. In old rats, STZ-treated animals had longer freezing time compared to the controls in the retention trials (24 h and 1 month later) but the difference was not statistically significant (repeated measures ANOVA, treatment effect: F(1, 20) = 4.08, *p* = 0.057; treatment*trial effect: F(2,40) = 3.06, *p* = 0.058). In young rats, there was no significant difference in retention trials either (Table 1A).

**Table 1:** Results of icv. STZ-injected (‘STZ’) and vehicle-treated (‘control’) rats in various behavioral assays. (A) Learning performance in the Fear conditioning paradigm. ‘Old rats’ column: + + + : *p* < 0.001 significant difference vs acquisition trial (post-hoc Duncan-test following repeated measures ANOVA with significant ‘trial’ effect (F(2, 40) = 18.36, *p* = 0.000). ‘Young rats’ column: +++: *p* < 0.001 significant difference vs acquisition trial (post-hoc Duncan-test following repeated measures ANOVA with significant ‘trial’ effect (F(2, 44) = 43.54, *p* = 0.000). Group size of old STZ-treated rats: n = 11 at retention trial 24 h and n = 10 at 1 month. (B) Passive Avoidance Learning. ‘Old rats’ column: +++: *p* < 0.001 significant difference vs acquisition trial (post-hoc Duncan-test following repeated measures ANOVA with significant ‘trial’ effect (F(1, 18) = 89.44, *p* = 0.000). ‘Young rats’ column: *: *p* < 0.05 significant difference vs control: unpaired t-test, t(20) = −2.56, effect size: 1.15; + + + : *p* < 0.001 significant difference vs. acquisition trial (post-hoc Duncan-test following repeated measures ANOVA with significant ‘treatment’ (F(1, 20) = 5.41, *p* = 0.030) and ‘trial’ (F(1, 20) = 397.41, *p* = 0.000) effects. Group size of old STZ-treated rats: n = 8. (C) Elevated plus maze results. ‘Old rats’ column: § *p* = 0.042 significant difference vs control (Mann–Whitney U-test, U = 29; because of variance inhomogeneity non-parametric test was used), effect size: 0.91. Group size of old STZ-treated rats: n = 10. Group size of young STZ-treated rats: n = 11.

		Old rats				Young rats			
	Test	Control		STZ		Control		STZ	
		Mean	± *SEM*	Mean	± *SEM*	Mean	± *SEM*	Mean	± *SEM*
A	FC acquisition trial freezing time (s)	31.7	± *14.39*	43.3	± *11.24*	123.0	± *11.95*	84.9	± *14.22*
	FC retention trial freezing time 24 h (s)	76.9^+++^	± *21.63*	161.2^+++^	± *30.52*	204.5^+++^	± *21.02*	208.4^+++^	± *22.15*
	FC retention trial freezing time 1 months (s)	89.0^+++^	± *25.22*	168.3^+++^	± *32.49*	187.0^+++^	± *25.74*	190.3^+++^	± *22.74*
B	PAL acquisition trial entry latency (s)	33.5	± *8.23*	33.3	± *2.86*	45.7	± *9.92*	88.6*	± *13.91*
	PAL retention trial entry latency (s)	226.1^+++^	± *35.06*	273.8^+++^	± *7.32*	278.4^+++^	± *17.07*	300^+++^	± *0*
	Not entered/total number of animals	8/12		6/8		10/12		10/10	
C	EPM time spent in open arms (s)	5.3	± *3.25*	34.1	± *17.98*	19.4	± *8.40*	12.2	± *5.0*
	EPM percentage of open/total entries	3.7	± *0.022*	21^§^	± *0.095*	19	± *0.063*	29	± *0.085*

### Novel object recognition (NOR)

**Figure 5:**
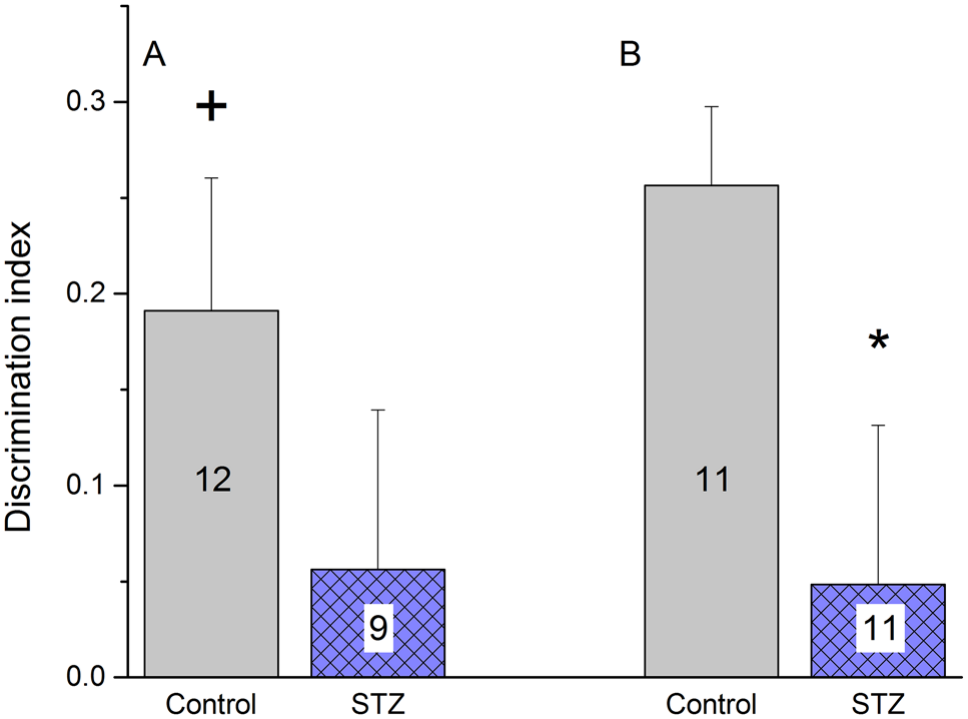
Novel object recognition performance of icv. STZ-injected (‘STZ’) and vehicle treated (‘control’) rats at Week 11 post-injection. Columns show means ± SEM values of discrimination index. Numbers inside the columns indicate the number of animals. (**A**) Results of old animals. + : *p* < 0.05 vs zero, singe sample t-test, control: t(11) = 2.76, STZ: t(8) = 0.67, ns.), effect size: 0.55.Three animals in the STZ group died before the test was carried out (**B**) Results of young animals. *: *p* < 0.05 vs control, unpaired t-test, t(20) = 2.24), effect size:0.96. Two rats were excluded from the evaluation according to the criteria described in the Methods section.

We found significant difference between the groups in the DI parameters in both experiments. In old rats, control animals showed a DI (0.19) significantly different from zero (no discrimination) whereas the DI of STZ-treated rats (0.06) did not differ from zero (Fig. 5A). In young rats, STZ-treated animals had a significantly lower DI (0.05) compared to the controls (0.25) (Fig. 5B).

### Pairwise visual discrimination in old rats

**Figure 6:**
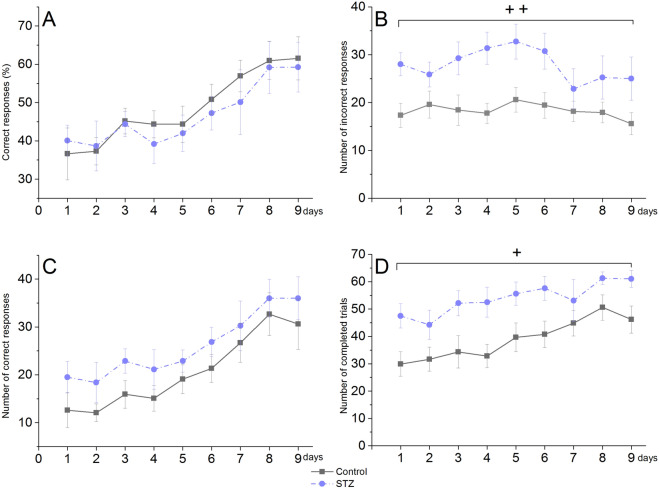
Pairwise visual discrimination performance of icv. STZ-injected (‘STZ’) and vehicle-treated (‘control’) old rats in a touchscreen apparatus in the post-injection period of Week 12–14. Means ± SEM values are shown. (**A**) Percentage of correct responses (**B**) Number of incorrect responses. + + : *p* = 0.005 significant treatment effect (F(1,18) = 10.28). (**C**) Number of correct responses. (**D**) Number of completed trials. + : *p* = 0.018 significant treatment effect (F(1,18) = 6.83). Group size of old STZ-treated rats: n = 8.

The STZ-treated animals made a significantly higher number of incorrect responses (Fig. 6B) and their number of completed trials were also significantly higher compared to the controls (Fig. 6D). Nevertheless, there was no difference between the two groups in the percentage and number of correct responses (Fig. 6A,C).

### Passive avoidance learning (PAL)

In old animals, there was no significant difference between the learning performances of groups either in acquisition or retention trials (Table 1B). In young animals, during the acquisition trial, the STZ-treated animals showed significantly longer latency to enter the dark chamber compared to the controls. In turn, there was no significant difference between the groups in the retention trial (Table 1B).

### Elevated plus maze (EPM)

In old rats, STZ-treated animals spent more time in the open arms and the ratio of open/total entries was significantly larger compared to the controls (Table 1C). In young rats, there was no significant difference between the two groups in either of the parameters (Table 1C).

### Open field (OF)

STZ-treated rats demonstrated significantly increased activity in both experiments. Consequently, they spent significantly less time in immobility (Table 2).

**Table 2:** Open field results of icv. STZ-injected (‘STZ’) and vehicle-treated (‘control’) rats.

Test	Old rats				Young rats			
	Control		STZ		Control		STZ	
	Mean	± *SEM*	Mean	± *SEM*	Mean	± *SEM*	Mean	± *SEM*
Ambulation time	188.4	± *23.23*	268.5*	± *23.62*	332.6	± *13.67*	409.2**	± *20.48*
Local movement time	727.9	± *34.40*	871.3*	± *38.34*	645.6	± *15.43*	758.5***	± *23.12*
Immobility time	833.4	± *55.32*	617.8*	± *49.67*	670.1	± *24.79*	541.4**	± *34.13*

Columns include means ± SEM values. ‘Old rats’ column: *: *p* < 0.05 significant difference between groups, unpaired t-test, ambulation time: t(18) = −2.32, effect size: 1.12, local movement time: t(18) = −2.72, effect size: 1.31, immobility time: t(18) = 2.72, effect size: −1.31. ‘Young rats’ column: **, ***: *p* < 0.01, *p* < 0.001 significant difference between groups; unpaired t-test, ambulation time: t(22) = −3.11, effect size: 1.33, local movement time: t(22) = −4.05, effect size: 1.73, immobility time: t(22) = 3.05, effect size: −1.30. Group size of old STZ-treated rats: n = 8.

### Phospho-tau and beta-amyloid levels

**Figure 7:**
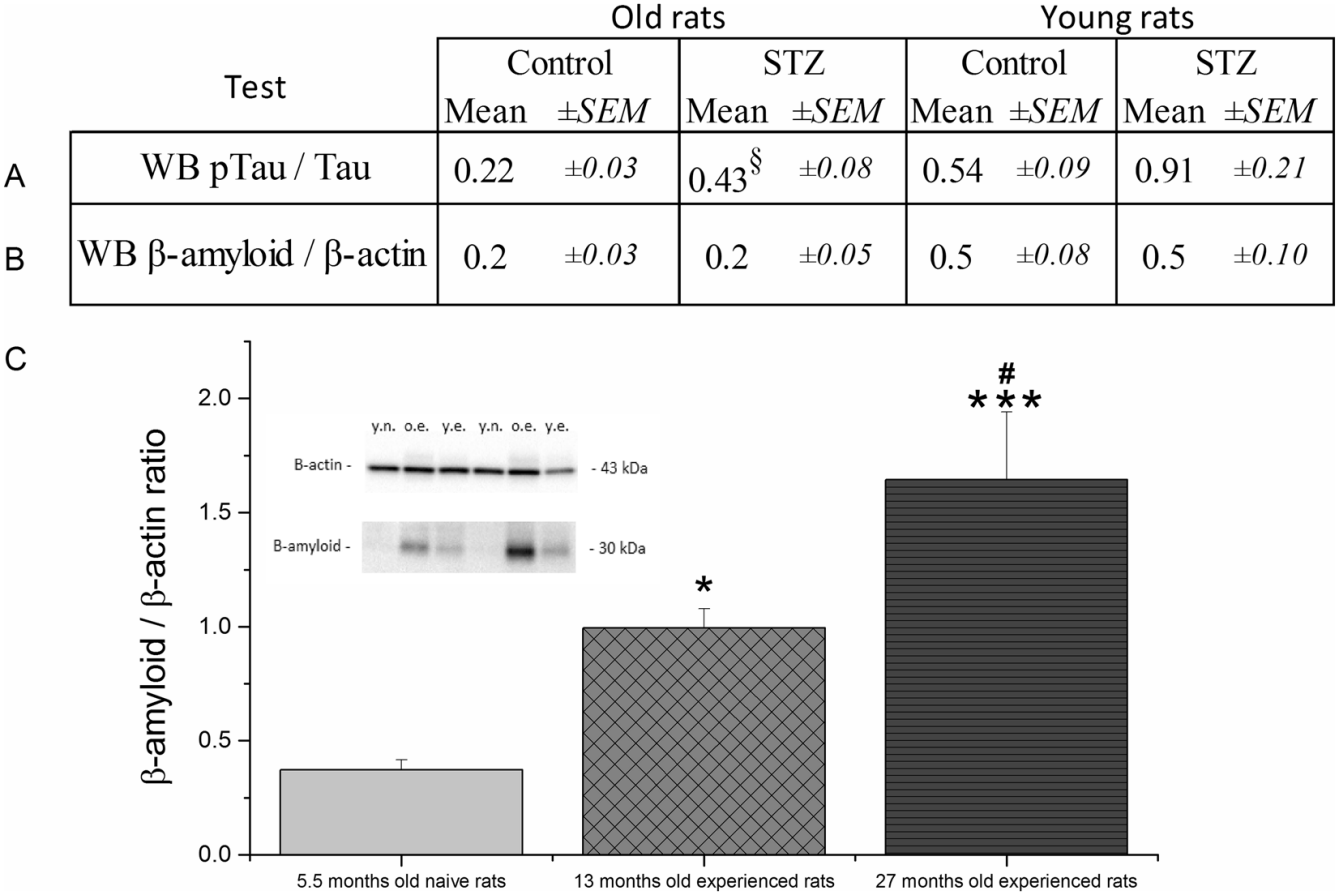
Results of the western blot assays. (**A**) Phospho-tau/tau ratio. ‘Old rats’ column: §:p = 0.016 significant difference vs control ((Mann–Whitney U-test U = 18; because of variance inhomogeneity non-parametric test was used), effect size: 1.23. Group size of old STZ-treated rats: n = 9. (**B**) β-amyloid level ns. Group size of old STZ-treated rats: n = 10. (**C**) Comparison of tissue protein levels of β-amyloid in 5 month old (young naïve), 12 month old (young experienced) and 25 month old (old experienced) rats measured by western blot. Means ± SEM values are shown. *, ***: *p* < 0.05, *p* < 0.001 significant difference vs. young naïve rats, #: *p* < 0.05 significant difference vs. young experienced rats (post-hoc Duncan test following one way ANOVA (F(2, 24) = 10.09, *p* = 0.001). Group sizes are 9, 11 and 11 for young naïve (y.n.), young experienced (y.e.) and old experienced rats (o.e.), respectively. The inset shows representative blots; original complete blots are presented in Supplementary material, Figs. S2-S3.

Significant elevated p-tau/tau ratio was found in old but not in young STZ-treated rats compared to their respective controls (Fig. 7A). There was no difference in β-amyloid levels between STZ-treated and control groups in either experiments (Fig. 7B). In a separate measurement we re-assayed the β-amyloid level in the young and old experienced control rats in parallel with the samples of naïve control young animals of 5 months age studied in our previous experiment^16^.We found an age-dependent increase in β -amyloid level with significant differences between the three age groups (Fig. 7C).

## Discussion

In young animals, STZ-treatment impaired recognition (NOR) and spatial memory (MWM) and attention (5-CSRTT). However, the latter effect was transient, as it passed by the end of the experiment suggesting that the previously acquired knowledge could compensate the detrimental effect. Impaired procedural memory (pot jump test) was also found in young STZ treated rats. In contrast, there was no significant difference between the control and STZ-treated groups in the PAL and FC tests, and in the cooperation paradigm; that is, STZ treatment did not affect fear memory and social learning.

STZ treatment increased novelty-induced exploratory activity in the open-field, but caused no significant difference in the anxiety levels of animals in the EPM. Biochemical markers, such as hippocampal β-amyloid and phospho-tau levels did not show significant differences either.

Looking at the results obtained in the old groups, first of all, 3 × 1.5 mg/kg STZ was more toxic to the old than to the young animals, as we lost four drug-treated rats during the post-treatment period. In old animals, similarly to young ones, STZ treatment impaired recognition (NOR) and spatial memory (MWM). However, in contrast to young rats, attention was not influenced by the treatment suggesting that the knowledge accumulated over the years became resistant to the impairing intervention. Procedural memory was also not affected, although this finding may have resulted from a floor effect, as old rats moved much shorter distances than young rats in the pot-jumping test. Social memory could not be evaluated due to mortality and thus disintegration of pairs. Fear memory was not affected in the PAL test, and—strictly in statistical terms—neither was it in the FC test. However, STZ treated rats showed about twice as much freezing as the controls during the retention trials. It may reflect better fear memory, however (1) it would be a surprising effect of STZ and (2) is not supported by the PAL results. A major difference between the PAL and FC paradigm is that in the former the animal has control over the situation (it may choose not to enter the dangerous place) while in the latter it has not (the rat is placed into the dangerous place) and as an anticipatory reaction to the imminent danger it shows freezing. Thus, the intensity of freezing reflects not only the strength of the memory trace but also the level of anxiety related to the previously experienced shock. In the pairwise visual discrimination task the two groups showed similar learning efficiency (% correct responses) although STZ-treated rats initiated and completed a significantly greater number of trials. Results of this assay suggests that the rats’ ability to acquire new knowledge was not disrupted by icv. STZ.

A peculiar and notable finding in the STZ-treated group was the increased percentage of premature responses in the 5CSRTT. This effect is interpreted as a sign impulsivity^27^. STZ treatment increased novelty-induced exploration in the open-field. Furthermore, rats from this group showed signs of decreased anxiety in the EPM test. The above results, together with the observed differences in the FC and pairwise discrimination tests, suggest that beside its cognitive effects icv. STZ exerted emotional effects as well. We interpret these findings as the compound elevated impulsivity in old rats. With this assumption, the seemingly contradictory results of the FC and EPM tests (increased vs decreased anxiety) may be explained as similar but context-dependent overreaction to the actual situation: in positive context (EPM) more courageous behavior, in negative context (FC) more fearful behavior. Also, the increased number of initiated trials in the pairwise discrimination paradigm may be interpreted as increased “interest” in the rewarded new task (positive context).

STZ differentially affected β-amyloid and phospho-tau levels: in the former no change could be observed while in the latter a significant increase was detected in the old rats.

Our results partly coincide with (MWM, NOR, phospho-tau) partly deviate (PAL, β –amyloid) from those published on young, albino, unexperienced rats.

Decreased spatial learning and memory performance in the MWM is one of the most common and characteristic effects of STZ experiments^39–55^ although in some studies the impairment was only observed in the probe trial^49,56,57^. The paper of Majkutewitz et al.^53^ is of particular relevance in this comparison as they—similarly to us—examined 22 months old rats and applied a protocol where the platform location changed day by day. Interestingly, in our previous study in young naïve Long-Evans rats^16^ we did not find impaired MWM learning.

Impaired recognition memory in the NOR test was also detected in several studies^16,52,58–62^.

Besides MWM, PAL impairment is the most common finding in the icv. STZ literature^7,40,43,44,50,54,58,59,63–67^. However, neither in this study nor in our previous experiment^16^ we could detect changes in this assay.

We found only one study^60^ where the effect of icv. STZ was investigated in the FC paradigm. The authors found decreased freezing response in the tone-conditioned but not in the context-conditioned version of the test.

Our findings of increased activity in the open-field are similar to those of Chen et al.^68^ and Guo et al.^56^ but in contrast to those of others who did not find difference in this test^54,63,64,66,67^.

Anxiety level of STZ-treated animals in the EPM was measured in two studies; Ileva et al.^69^ observed—in contrast to our results—increased anxiety in young STZ-treated animals, while Moreira-Silva et al.^60^ found no difference from the control.

Elevated β -amyloid level is a common finding in the literature^16,44,48,58,61,62,69–72^, however it was not confirmed in the present study. As in the cited studies typically 4–6 months old rats were used, a possible explanation for this discrepancy may be that the 12 and 25 months old animals of the current study already had high protein levels resulting in a ceiling effect in the STZ treatment. This assumption is backed up by our finding of a significant age-dependent increase in β -amyloid level showing appr. threefold higher levels in the 12 months old than in the 5 months old rats. For comparison: STZ could cause a 2.2 fold increase in the amount of β-amyloid in the 5 months old rats in our previous study^16^. However, as our 12 and 25 months old rats also showed cognitive impairment, the above finding suggest that the eventual effect of STZ on β -amyloid formation may not be a causative factor in its detrimental cognitive effects.

Increased phospho-tau/tau ratio was also reported in many studies^16,43,47–49,51,56,58,60–62,72^. In the current study we only detected a significant increase in the old animals, while in young rats a non-significant 68% increase was observed. Interestingly, Osmanovic Barilar et al.^73^ examined STZ-treated rats of different ages and found increased phospho-tau/tau ratio in 6 and 9 months old rats but not in 12 months old ones.

Impulsive-like behavior has not been described in the literature yet, and it may be a hint for a possible direction of further investigations. It is not among the characteristic non-cognitive symptoms of AD^74,75^, rather, impulsivity and disinhibition are well known symptoms of frontotemporal dementia^75–77^, which lacks amyloid pathology^78,79^.

Comparison of our results with those in the literature shows that the effect of icv. STZ varies in different strains, depends on the age of animals and influenced by their level of experience and learning history. However, if the method is to be considered as a dementia model then the translationally most relevant animal population should be that of (i) old and (ii) experienced rats. Up to our best knowledge the present study is the first where the effect of icv. STZ was investigated in such a population. In these animals icv STZ produced impairments in spatial and recognition memory but not in fear learning/memory, visual discrimination and social learning; however it induced impulsive-like behavior. β-amyloid level was not increased probably because of the high basal level.

Nevertheless, it would be premature to generalize these findings to the STZ-icv model as such, since beside the age and experience level of the animals several other factors differed from those common in the literature. Strain difference is one of them: Long-Evans rats are better performers in cognitive tasks than Wistar rats^11–15^, and there is also a difference in local cerebral blood flow reactivity^80^. The dose of STZ applied in our study (4.5 mg/kg)^16^ was greater than those used in the literature (not greater than 3 mg/kg) and we do not know whether the Alzheimer disease-like pathophysiology induced by 3 mg/kg and 4.5 mg/kg STZ-icv has the same time course of onset, development and progression in Long-Evans and Wistar rats. Last, cognitive performance is usually measured in ad libitum fed rats, while we applied a food restriction regime, which may have rendered the animals more resistant to the toxic effects of STZ^18,21,22^. Thus, findings of the current study together with the above discussed differences call for more extensive studies with the STZ model involving both Wistar and Long-Evans strains to further strengthen and specify its translational validity.

## Supplementary Information

Supplementary Information.

## Data Availability

The raw data supporting the conclusions of this article will be made available by the authors, without undue reservation. To request data from this study, please contact the corresponding author.
