# Supplementary material for: Performance of the intracerebroventricularly injected streptozotocin Alzheimer’s disease model in a translationally relevant, aged and experienced rat population
Source: Sci Rep. 2022 Nov 24;12:20247. doi: 10.1038/s41598-022-24292-5 (PMC9691696; doi:10.1038/s41598-022-24292-5)
Supplement: Supplementary file 1 — Supplementary Information. [file 41598_2022_24292_MOESM1_ESM.docx]

**Performance of the intracerebroventricularly injected streptozotocin Alzheimer’s disease model in a translationally relevant, aged and experienced rat population**

Attila Gáspár, Barbara Hutka, Aliz Judit Ernyey, Brigitta Tekla Tajti, Bence Tamás Varga, Zoltán Sándor Zádori, István Gyertyán*

Department of Pharmacology and Pharmacotherapy, Semmelweis University, Budapest, Hungary

***Corresponding author:**

István Gyertyán

[gyertyan.istvan@med.semmelweis-univ.hu](mailto:gaspar.attila@med.semmelweis-univ.hu)

1089, Nagyvárad tér 4, Budapest, Hungary

T: +36208256560

**Materials and methods**

Supplementary data:

*Animals*

Subjects weighed 370–470 g in both experiments, were held in 1376 cm^2^ polycarbonate cages and fed with commercial pellet (SM Rat/Mouse produced by SSniff Spezialdiäten GmbH, Soest, Germany)

*Intracerebroventricular streptozotocin treatment*

Rats were anaesthetized via a mixture of ketamine (80 mg/kg) (Produlab Pharma B.V. Raamsdonksveer, Netherlands) and xylazine (10 mg/kg ip.) (Produlab Pharma B.V. Raamsdonksveer, Netherlands) during the first drug administration and isoflurane (4% in pure oxygen) (CP-Pharma GmbH, Burgdorf, Germany) during the 2^nd^ and 3^rd^ surgeries. We changed the anesthetic because the second and third operations were much shorter than the first, and we did not want to repeatedly expose our animals to (unnecessarily) long ketamine-xylazine anesthesia, which would have imposed more physiological stress and carried bigger safety hazard, especially for old rats. Animals were placed in a stereotactic apparatus (Stoelting, Wood Dale, IL, United States) and laid on a heating bench (37°C) (Supertech Instruments, Pécs, Hungary). Midline incision on the skin was made and the surface of the skull was cleaned. Drilled holes at the place of the injection was made by dental drill. The icv. coordinates were: 0.72 mm posterior to bregma, 1.5 mm lateral to sagittal suture, 3.6 mm ventral of the surface of the brain^1^. A guide cannula was placed into the drilled hole in the skull and STZ was infused by a Hamilton syringe via a microinjection pump (CMA/100, CMA/Microdialysis Ab, Stockholm, Sweden); the injection speed was 5 min/hole. The needle was left in place for an additional 2 minutes then the guide cannula was removed and the wound sutured. After the last treatment, the holes were closed by bone-cement. After the surgery, rats were given buprenorphine (0.05 mg/kg i.p.) (Richter Pharma AG, Wels, Austria) and lidocaine (Egis Pharmaceuticals, Budapest, Hungary) was applied to the wound as analgesics. Until the wounds healed (approximately two weeks), the animals were kept separately.

*Behavioral assays*

Morris water maze (MWM)

Rats learned the task at the age of 3 months in 4 days with the platform fixed at the SE quadrant, then they received maintenance training sessions once a month until the start of the experiments. A maintenance training session consisted of 3 trials, and the location of the platform was rotated around the four quadrants from session to session. During the post treatment measurements, the animals were tested in four sessions at weeks 4, 6-8, 9-11 and 14-15 (old rats), and 4, 7, 10, 13 (young rats). Some of the old animals had swimming difficulties and they had to be rescued before the hidden platform was found; these animals were given a latency time of 180 seconds (one rat at week 4, three at weeks 6-8, two at weeks 9-11 and three at weeks 14-15).

5-choice reaction serial time task (5CSRTT)

Rats were trained for the task in stages with gradually decreased stimulus duration from 30 to 1 s. Animals could step to the next training stage, if they collected at least 30 (old rats) or 40 rewards (young rats) during a training session. The behavior of the animals was recorded by the TSE Operant Behavior v03.07 software (TSE Systems, Bad Homburg vor der Höhe, Germany). Old rats learned the task from the age of 6 to 8 months while young rats acquired this knowledge from the age of 2 to 4 months, followed by maintenance training sessions once a week in both groups.

Pot jumping

Old animals got acquainted with the task at the age of 3 month and had training sessions with biweekly frequency further on. Young rats had an initial training period of 3 weeks with daily sessions starting at the age of 3 months then had practicing sessions once every week.

Cooperation task

Rats were trained for the task in increasing difficulty stages. In early stages, the animals were kept alone in the box, and they had to learn how to use the nose poke and lever press modules. The rats were learning through stages with gradually increasing the time they needed to hold their noses in the nose-poke module to receive a reward pellet. After this, they learned how to use the lever press module. If they successfully used these modules separately, in the next stage they learnt how to use them in sequence: a 3 sec long nose-poke - instead of yielding a pellet – activated the lever which had to be pressed in order to get the reward. After the rats learnt successfully this combined response, they were put together in pairs and had to work together to obtain the reward pellets. Whichever animal made a nose-poke, it activated the other rat’s lever, which had to be pressed in order to get a pellet. Animals could step to the next training stage, if they collected at least 30 pellets during a training session. Programming the equipment was done by MED-PC IV v4.2 software (MedAssociates, VT, USA). Rats started to learn the task at the age of 3 (old rats) or 4 (young rats) months, and 9-12 weeks were needed to acquire it. This period was followed by regular maintenance training sessions once or twice a week.

Novel object recognition (NOR)

The objects were placed 10 cm from the diagonally opposite corners and 40 cm from each other. No habituation trial was performed because three months (in case of old animals) or ten months (young animals) before the test, a preliminary NOR test with different recognizable objects was already performed with the animals.

Pairwise visual discrimination

Animals were pre-trained for this task with increasing difficulty levels consisting of 5 stages. A stage was completed when the rat gain 20 pellets. The whole conditioning procedure took place from Week 12 to Week 14 post-injection. During the task, image projection and nose poke detections was controlled by WhiskerServer v4.0.0 control system (Cambridge University Technical Services Ltd., Cambridge, UK).

*Western blot (WB)*

Hippocampal tissues were homogenized with TissueLyser (Qiagen, Venlo, Netherlands) in lysis buffer containing 200 mM NaCl, 5 mM EDTA, 10 mM Tris, 10% glycerine, and 1 g/ml leupeptin (pH 7.4), supplemented with a protease inhibitor cocktail (cOmplete ULTRA Tablets, Roche, Basel, Switzerland) and PMSF (Sigma, St. Louis, MO, United States). The homogenized lysates were centrifuged twice at 1,500x g and 4°C for 15 min, then the supernatants were collected and their protein concentration was measured by the bicinchoninic acid assay (Thermo Fisher Scientific, Waltham, MA, United States). Equal amount of protein (20 μg) was mixed with Pierce Lane Marker reducing sample buffer (Thermo Fisher Scientific, Waltham, MA, United States), and loaded and separated in a 4–20% precast Tris-glycine SDS polyacrilamide gel (Bio-Rad, Hercules, CA, United States). Proteins were transferred electrophoretically onto a polyvinylidene difluoride membrane (Bio-Rad, Hercules, CA, United States) at 200 mA overnight. Membranes were blocked with 5% nonfat dry milk (Cell Signaling Technology, Leiden, Netherlands) in Tris buffered saline containing 0.05% Tween-20 (0.05% TBS-T; Sigma, St. Louis, MO, United States) at room temperature for 2 h. Membranes were incubated with primary antibodies against Phospho-Tau (p-tau) (PHF-13, sc32275, 1:1,000, Santa Cruz Biotechnology, Santa Cruz, CA, United States), Tau (sc32274, 1:1,000, Santa Cruz Biotechnology, Santa Cruz, CA, United States) and β-Amyloid (sc28365, 1:1000, Santa Cruz Biotechnology, Santa Cruz, CA, United States) overnight at 4°C, followed by 2 h incubation at room temperature with anti-mouse HRP-linked secondary antibody. Phospho-Tau protein expression was normalized to the corresponding total protein. β -actin was used to control for sample loading and protein transfer and to normalize the content of the β-amyloid. Signals were detected with a chemiluminescence kit (Bio-Rad, Hercules, CA, United States) by Chemidoc XRS+ (Bio-Rad, Hercules, CA, United States). The intensity of the samples was measured by Image Lab software (version 4.1, Bio-Rad, Hercules, CA, United States). Phospho-specific antibody was removed with Restore™ Western Blot Stripping Buffer (Thermo Fisher Scientific, Waltham, MA, United States) before the incubation of the corresponding total protein antibody.

**Results**

Cooperation

In old rats, because of the high mortality rate the pairs were broken and it was not possible to evaluate the data. In young rats, there was no significant difference between the learning performances of the two groups (Fig S1).





**Figure S1.** Learning performance of icv. STZ-injected (‘STZ’) and vehicle-treated (‘control’) young rats in a cooperation task at various time points post-injection. Means ± SEM of number of rewarded trials are shown. n=6 pairs in each group

Western blots of beta-amyloid level determination in vehicle-treated control animals of 3 experimental groups (background data for Figure 7).


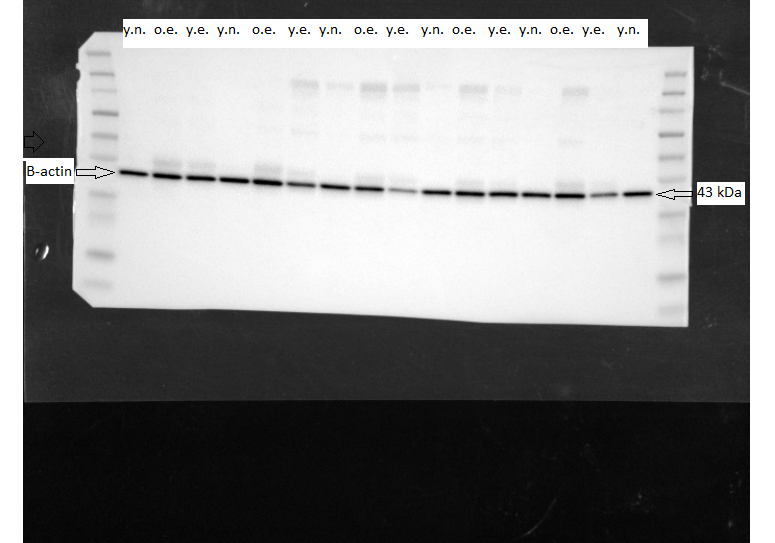


**Figure S2.** Western blot measurements of β-actin levels. Young naïve (y.n.), young experienced (y.e.) and old experienced rats (o.e.)


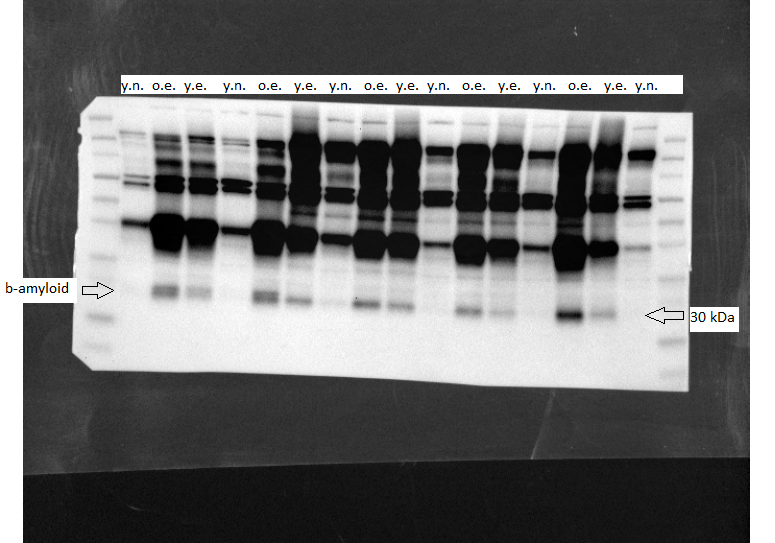


**Figure S3.** Western blot measurements of β-amyloid levels. Young naïve (y.n.), young experienced (y.e.) and old experienced rats (o.e.)

**References**

1. Noble, E. P., Wurtman, R. J. & Axelrod, J. A simple and rapid method for injecting H3-norepinephrine into the lateral ventricle of the rat brain. *Life Sci.* **6**, 281–291 (1967).
